# The cancer-testis antigen, sperm protein 17, a new biomarker and immunological target in head and neck squamous cell carcinoma

**DOI:** 10.18632/oncotarget.22213

**Published:** 2017-10-31

**Authors:** Christopher A. Schutt, Leonardo Mirandola, Jose A. Figueroa, Diane D. Nguyen, Joehassin Cordero, Klauss Bumm, Benjamin L. Judson, Maurizio Chiriva-Internati

**Affiliations:** ^1^ Division Otolaryngology, Department of Surgery, Yale School of Medicine, New Haven, CT, USA; ^2^ Kiromic, Inc., Houston, TX, USA; ^3^ Department of Otolaryngology (ENT), TTUHSC, Lubbock, TX, USA; ^4^ CaritasKlinikum Saarbrücken, Saarbrücken, Germany; ^5^ Department of Multiple Myeloma & Lymphoma, University of Texas, MDACC, Houston, TX, USA

**Keywords:** cancer/testis antigens, head and neck squamous cell carcinoma, immunotherapy, biomarker, sperm surface protein 17

## Abstract

Head and Neck Squamous Cell Carcinoma is a deadly and locally aggressive malignancy that frequently portends a poor prognosis. Since current treatment modalities including surgery, chemotherapy and radiation are heavily debilitating and often result in recurrence intense efforts have been put into the development of novel less toxic and more lasting treatment strategies. Recently, immunotherapy has been proposed as a promising alternative that could potentially meet these requirements.

SP17 is a validated cancer-testis antigen in multiple myeloma, ovarian cancer and non-small cell lung cancer. We aim at studying SP17 expression in HNSCC and its immunogenicity as a possible future target for HNSCC therapeutic vaccines.

SP17 expression was evaluated in tissue specimens of HNSCC patients and controls. Moreover, SP17 immunogenicity was studied by generating autologous dendritic cells *in vitro* from the peripheral blood mononucleated cells of HNSCC patients and testing their ability to induce SP17 specific cytotoxic lymphocytes capable of killing autologous tumor cells *in vitro*. SP17specific immune responses were also evaluated in HNSCC patients as circulating anti-SP17 autoantibodies.

SP17 was expressed in HNSCC tissues of HNSCC patients. Autologous dendritic cells pulsed with SP17 antigen induced powerful SP17 MHC class-I restricted, perforin-dependent, cytotoxic T-cells capable of efficiently killing autologous tumor cells *in vitro*. SP17-specific autoantibodies were detectable in the serum of HNSCC patients irrespective of tumor site or TNM stage.

In conclusion, SP17 is an ideal immunotherapeutic target for HNSCC and a potential serological biomarker of the disease.

## INTRODUCTION

Head and neck squamous cell carcinoma (HNSCC) is the sixth most common cancer worldwide. In the USA, it accounts for more deaths annually than cervical cancer, malignant melanoma or Hodgkin's lymphoma. It's estimated annual global incidence is greater than 500,000 cases, with 17 newly diagnosed tumors per year per 100,000 people.

Aggressively arising from the mucosa of the upper aero-digestive tract, HNSCC is classified by sub-site, which include the oral cavity, oropharynx, nasopharynx, hypopharynx and larynx [1, 2]. Presently, treatments options for HNSCC are limited. They consist of surgery, chemotherapy, and radiation given as solo-agents or in combination. Unfortunately, these treatments have well-known toxicities, affecting the function of normal deglutition, phonation, and respiration and regularly resulting in major cosmetic disfiguration. Despite the aggressiveness of such approaches, the 5-year survival rates have not significantly improved over the last three decades. Thus, the development of novel therapeutic strategies against HNSCC appears a matter of urgency. One promising strategy is immunotherapy, which is more specific than conventional treatment and induces memory responses that could yield long-term tumor immune-surveillance and reduce the incidence of relapses, thus increasing long-term disease-free survival [2]. In the quest for the development of immunotherapy approaches for cancer, the discovery and validation of specific targets is critical. We have previously shown that SP17 (Sperm Protein 17) is an ideal target for ovarian cancer, multiple myeloma, and non-small cell lung cancer immunotherapy [3–6]. Moreover, we recently found that circulating anti-SP17 antibodies are detectable in the serum of non-small cell lung cancer patients, supporting SP17 immunogenicity and vaccine strategies targeting this antigen. On these bases, we investigated and validated SP17 as a new biomarker and therapeutic target for HNSCC.

## RESULTS

### SP17 is expressed in HNSCC primary tumors

**Figure 1:**
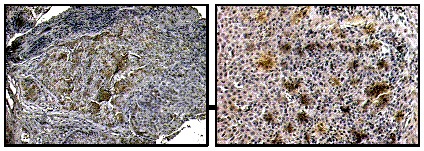
IHC for SP17 in HNSCC Representative results of IHC analysis for the expression of SP17. Brown signal from the HRP-DAB reaction indicates sites of SP17 expression. Left picture: carcinoma of the larynx (20X). Right picture: oropharynx carcinoma (40X).

Figure 1 shows representative IHC (Immuno-Histochemistry) results obtained on samples for HNSCC patients, diagnosed with carcinoma of the larynx (left picture) and of the oropharynx (right picture). On average, about 30% of the cells in the tumor tissue were SP17^+^. SP17 positivity status did not correlate with the grade, T/N stage, or tumor site.

### SP17 expression by HNSCC tumors elicits spontaneous humoral responses in patients

**Figure 2:**
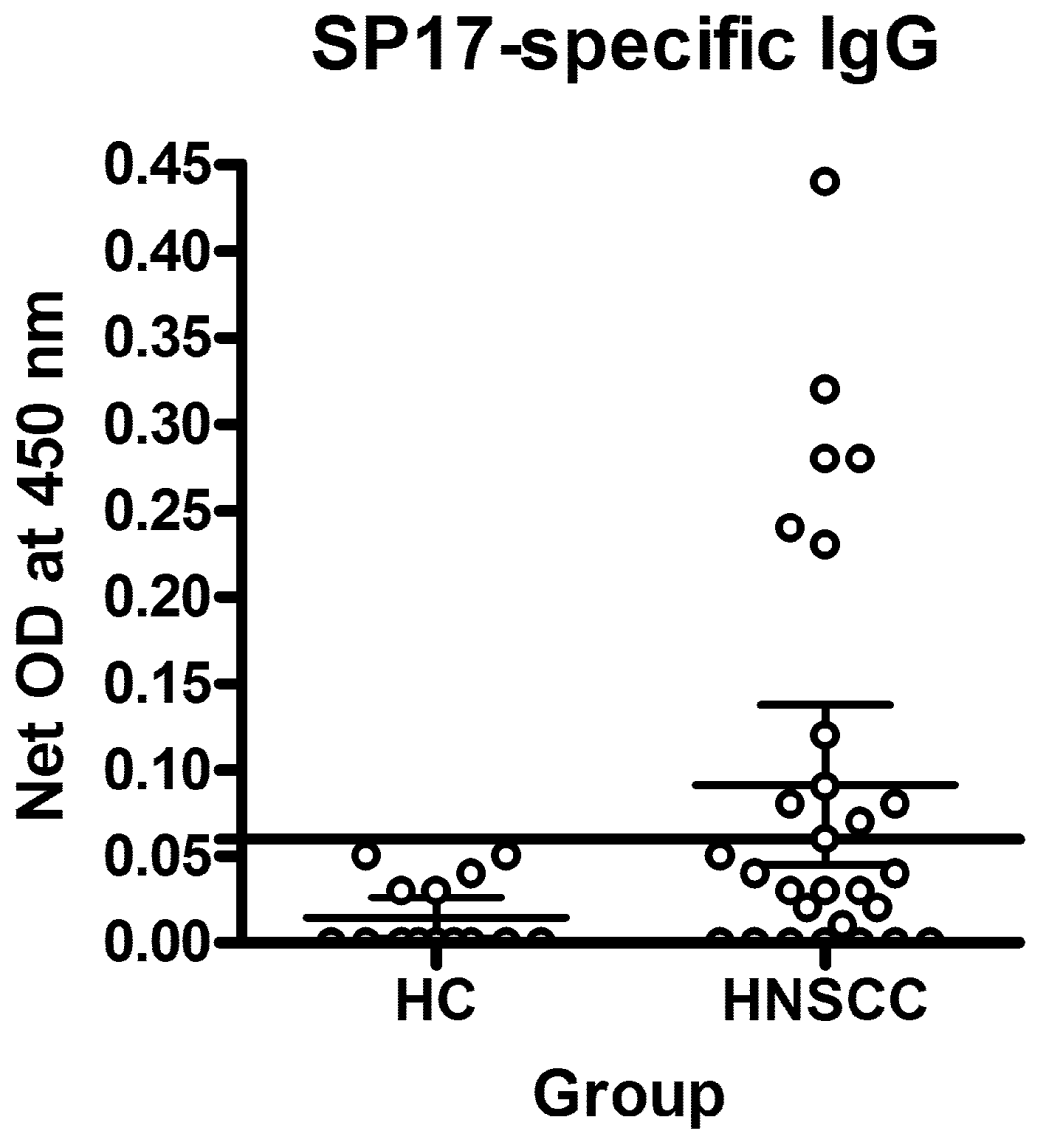
Analysis of SP17-specific circulating auto-antibodies Graphs display mean OD values calculated from experiments run in quadruplicate. The horizontal line represents the positivity cut-off.

To evaluate SP17 immunogenicity in HNSCC patients, we measured SP17-specific circulating auto-antibodies (IgG) by ELISA as means of experiments run in quadruplicate (Figure 2 and Table 1). The cut-off (horizontal line) was determined as described in Methods and it shows that 11 out of 35 subjects tested positives for SP17-specific auto-antibodies.

**Table 1:** SP17-seropositivity

Cohort	Number of SP17-seropositive samples (%)	Number of SP17-seronegative samples (%)
HNSCC	11 (31)	24 (68)
Healthy controls	0 (0)	30 (100)

### SP17-specific CTL kill autologous tumor cells but not SP17-negative target cells *in vitro* through a perforin-dependent mechanism

**Figure 3:**
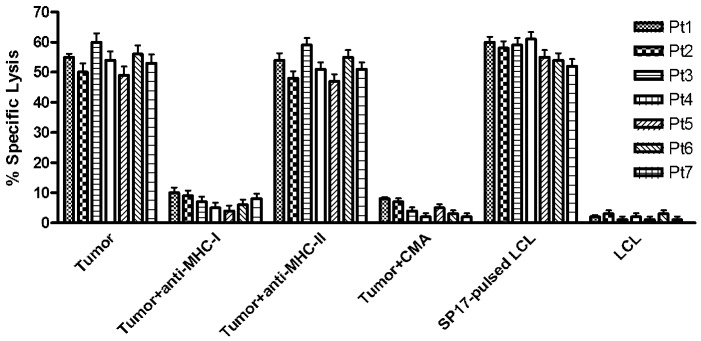
SP17-primed CTL lyse autologous tumor cells expressing SP17 Tumor cell cytotoxicity by CTLs could be blocked by a monoclonal antibody directed at monomorphic HLA-class I molecules but not HLA-class II molecules. Autologous tumor cell lysis by SP17-specific CTLs could be blocked by CMA, suggesting that target cell cytotoxicity was mediated via the perforin pathway. CTL were able to kill autologous Lymphoblastoid Cells Lines (LCL) in SP17-dependent manner when loaded with Sp17 protein as a positive control. CTLs were not able to kill LCL alone (SP17-negative). All results expressed as mean ± SD of triplicate experiments, with an effector:target ratio of 20:1.

The immunophenotype of autologous CTL (Cytotoxic T Lymphocyte) stimulated with SP17-presenting DC (Dendritic Cells) showed that they were predominantly composed by CD8+CD56- cells, indicating the expansion of CD8+ T-lymphocytes and the absence of CD56+ natural killer cells. CTL were able to efficiently kill autologous tumor cells and SP17-pulsed autologous LCL cell lines (Lymphoblastoid Cell Lines, specific lysis was∼ 58% on average for both targets), but not SP17-negative LCL cells (specific lysis was less than 5%). Moreover, the addition of MHC class I blocking antibodies significantly reduced the specific lysis of about 10 folds, whereas a MHC class II blocking antibody did not affect CTL activity. Since CMA (Concanamycin A) blocked CTL activity similarly to MHC class I blockade, we concluded that the mechanism of cytotoxicity was perforin-dependent (Figure 3).

## DISCUSSION

Our study demonstrates that select HNSCC tumor cells express the cancer-testis antigen SP17, that the antigen naturally invokes an immune response *in vivo*, and possesses immunogenicity capable of destroying cancer *in vitro*. These attributes allow SP17 to be an excellent target for the immunological treatment of this deadly disease. Moreover, we show that SP17 antibodies are found in the serum of HNSCC patients but not of healthy controls, suggesting that SP17 could be exploited as a serologic biomarker of this disease.

Traditional treatment of advanced HNSCC relies on surgery, radiation and chemotherapy. The combination of modalities depends on stage, site of disease and prognostic indicators of the tumor. Surgery often leaves anatomical deficits with impaired function, while chemo-radiotherapy anatomically spare structures but often leave them without function. [7–10]. Even with aggressive treatments such as radical tumor resection with post-operative chemo-radiotherapy, long-term disease control is hard to achieve in the majority of patients with advanced disease. As a result, aggressive treatments fail to extend the 5-year survival rates [11]. Such poor prognosis stems from the limited treatment options for advanced disease. To date, no molecular targeted drugs have been developed for HNSCC other than Cetuximab and Bevacizumab [12]. Given this poor clinical scenario, the development of novel treatments, such as immunotherapy, is needed.

Tumor-associated antigens (TAA) discovered and validated in HNSCC have been proposed as targets in peptide-based vaccines. The ultimate goal of such therapeutic cancer vaccines is to induce tumor regression by activating the adaptive immune response following the presentation of TAA in the context of a peptide–major histocompatibility complex (MHC). This in turn triggers T-cell recognition and cytotoxic T-cell (CTL)-mediated tumor cell killing. This strategy might be the key for a future cure or long-term control of HNSCC, as shown by the recent results by Yoshitake Y. *et al.* [13], who reported a significant correlation between extended overall survival of HNSCC patients and the T-cell specific responses to two CTA, namely LY6K and CDCA1, following a peptide vaccine therapy in a Phase II clinical trial. In this context, SP17 is potentially an ideal candidate for therapeutic cancer vaccines against HNSCC, because of its reported selective expression in malignant cells [3–6] and the high immunogenicity shown in this study. We have previously shown that a SP17 protein-based vaccine affords for tumor eradication and long-term protection in a murine model of ovarian cancer without inducing short or long-term toxicities [5]. This has been supported by a pilot human clinical trial evaluating a SP17-based therapeutic vaccine in subjects with multiple myeloma and ovarian cancer, in which SP17-specific immunological responses were not followed by vaccine-related toxicities [14]. Atanackovic D. *et al.* [1] found a number of CTA expressed in HNSCC at the mRNA level, namely MAGE-A3, SSX-1, MAGE-C2, MAGE-C1, BAGE, SSX-2, SCP-1, NY-ESO-1, and HOM-TES-85. Although these are potentially new CTA targets for HNSCC immunotherapies, no data is available at present about their expression at the protein level in tumor cells and their immunogenicity in terms of inducing specific CTL responses against HNSCC. Due to the high heterogeneity of this disease, it is likely that effective vaccines will include a panel of CTA target antigens [2], of which SP17 is an ideal candidate, considering i) its selective expression in tumor cells and ii) its high immunogenicity as shown by our results.

Human papilloma virus (HPV) is a relevant etiologic factor in the pathogenesis of oropharyngeal squamous cell carcinomas [15]. Consequently, anti-HPV vaccines have been proposed as an effective immunotherapeutic strategy. The HPV oncoproteins E6 and/or E7 have been successfully targeted in HPV^+^ HNSCC, as demonstrated by Voskens C. *et al.* [16] On this basis, FDA-approved anti-HPV vaccines such as Cervarix and Gardasil are worthy of careful evaluation to prevent HPV infections in HNSCC and as therapeutic strategies for patients with loco-regional recurrence and metastatic disease [2]. Noteworthy, we have found SP17 in HNSCC regardless of the HPV status. We hypothesize that SP17 could be especially intriguing as an immunotherapeutic target since it could represent an attractive alternative for patients with HPV-negative tumors.

Similarly to our previous study of SP17 in non-small cell lung cancer [6] and AKAP-4 in prostate cancer [17], we at first evaluated SP17 immunogenicity by detecting serum anti-SP17 autoantibodies in HNSCC patients. Notably, SP17 antibodies were found at a higher rate (about 31% of samples) compared with the anti-p53 autoantibodies previously described in 25% of HNSCC patients [18]. Most importantly, we found SP17 antibodies in the serum of subjects with regionally confined as well as distant (metastasized) disease, regardless of the location of the primary tumor. If validated in larger, independent studies, SP17 antibodies might be useful in the diagnosis and/or prognosis of HNSCC, alone or in combination with recently discovered serological biomarkers of the disease, namely IFN-γ, IL-13, and MIP-1β [19].

Our data shows that SP17-presennting dendritic cells (DC) can be generated *in vitro* and that they afford for the induction of powerful CTL responses against autologous tumor cells. DC cells are the immune cells with the most powerful antigen presenting capacity and are the only ones that can activate naïve T- and B-cell immune responses. In HNSCC, targeting the p53 protein has been widely investigated given the high incidence of these mutations in HNSCC. A Phase I clinical trial enrolling advanced HNSCC treated with 3 injections of DC pulsed with wild-type p53 peptides showed no grade 2–4 toxicity and 88% 2-year disease free survival [20].

Here we have proven that it is possible to generate SP17-pulsedd DC *in vitro* from the peripheral blood of HNSCC patients, and that such DC are capable of inducing potent MHC class-I and SP17-restricted tumor cell killing by autologous CTL. Therefore, we hypothesize that SP17 could represent an ideal DC vaccine target for HNSCC, since unlike p53, it is tumor-specific.

The role of the immune responses in the control, initiation, and progression of HNSCC is only starting to be appreciated. The active, SP17-specific immune response we observed *in vitro* pinpoints that the expression of SP17 by HNSCC might be therapeutically relevant and serve as the rationale for implementing new immunotherapeutic treatments. We hypothesize that DC manipulated *in vitro* to present SP17 may trigger the activation of Th1-polarized CTLs capable of overcoming the immunosuppression seen in the HNSCC [21, 22], once adoptively transferred to patients. Further studies are required to determine the prevalence of SP17 expression in the HNSCC patient population, and to optimize the process of generating SP17-specific CTL responses *in vivo* overcoming the tumor-initiated immune suppression. Specifically, we foresee that SP17-targeted DC vaccination or autologous SP17-specific CTL transfer will most likely not be effective as a mono-therapy, due to the suppressive microenvironment typical of HNSCC [23]. On this regard, PD1/PD-L1 blockers are promising adjuvant drugs [24], and accordingly in August 2016 the FDA approved pembrolizumab (Keytruda^®^) for the treatment of patients with an advanced form of head and neck cancer. Following this approval, the KEYNOTE-040 trial evaluated the efficacy of pembrolizumab versus standard of care (i.e. methotrexate, docetaxel, cetuximab) for recurrent or metastatic HNSCC (clinical trial identifier NCT02252042). In July 2017, it was announced that the trial did not meet its primary endpoint of improved overall survival. Many hypotheses can be made to explain KEYNOTE-040 failure, but one critical point is that no active immunotherapy, such as a cancer vaccine, was given in combination with pembrolizumab. In this scenario, we reason that the while that treatment released the T-cell dysfunction caused by tumor up-regulation of PD-L1, there was a lack of concomitant T-cell activation which would be required to generate anti-tumor CTLs. Therefore, we warrant future studies combining an SP17-DC vaccine or adoptive transfer of SP17-specific CTLs generated *in vitro* with PD1/PD-L1 blockers.

## MATERIALS AND METHODS

### Tumor, serum and peripheral blood samples

Samples consisted of 24 tissue samples from primary HNSCC (Table 2), 35 serum samples of HNSCC and 30 serum samples from healthy controls (Table 3), and 7 peripheral blood samples from HNSCC patients. Patients' material was collected at the Friedrich-Alexander University Medical School, University of Erlangen-Nurnberg, Erlangen-Nurnberg, Germany, at the Section of Otolaryngology, Yale University School of Medicine, New Haven, Connecticut, and at the Texas Tech University Health Sciences Center, Lubbock, TX, USA.

**Table 2:** Characteristics of the HNSCC tissue samples cohort

Characteristic	Number of samples (total=24)
T0	2
T1	4
T2	5
T3	8
T4	5
N0	8
N1	3
N2	11
N3	2
Hypopharynx	5
Larynx	7
Nasopharynx	5
Oropharynx	7

**Table 3:** Characteristics of the serum samples cohorts

Characteristic	Number of samples
HNSCC (median age= 61 years)	35
*T status*	
T1	7
T2	15
T3	6
T4	7
*N status*	
N0	14
N1	8
N2	9
N3	4
*Tumor site*	
Oral cavity	16
Larynx	7
Oropharynx	12
Healthy controls (median age=40 years)	30

### Immunohistochemistry (IHC)

Tissues were embedded in paraffin and 3 μm thick-sections were prepared. Slices were exposed to the anti-SP17 primary antibody (anti-SP17 mouse monoclonal antibody that we have previously described [25]) and then incubated for 30 minutes with the HRP-linked secondary antibody (Santa Cruz Biotechnology, 1:500 dilution in PBS/BSA 0.1%) and 5 minutes with DAB (3,3'-diaminobenzidine, DAKO, Glostrup, Denmark). Pictures were taken at 10X, 20X, 40X and 63X objective magnifications using a DMI3000 B inverted microscope (Leica Microsystems GmbH, Wetzlar, Germany) and analyzed by the Leica Application Suite (LAS) software (Leica Microsystems GmbH).

### ELISA for SP17-specific auto-antibodies

The measurement of SP17-directed IgG in the serum of HNSCC patients and heathy controls was evaluated as we have recently described [6, 25]. The positivity cut-off was set in accordance to the Clinical and Laboratory Standards Institute's (CLSI) guidelines, using MedCalc software.

### Generation of SP17-pulsed dendritic cells (DC)

PBMCs were separated from heparinized venous blood by Ficoll-Hypaque (Sigma) density gradient centrifugation. DCs were generated from peripheral blood monocytes as described previously [26]. Briefly, PBMCs were seeded into six-well culture plates containing 3 mL of RPMI-1640 and 10% Fetal Calf Serum (FCS) at 5-10 x 10^6^ per well. After 2 hours at 37 °C, non-adherent cells were removed and the adherent cells were cultured at 37 °C in RPMI-1640 supplemented with 10% FCS, 800 IU/ml GM-CSF (Immunex, Seattle, Washington) and 1000 IU/ml IL-4 (Genzyme, Cambridge, USA). After 7 days of culture, DC were harvested for pulsing with SP17 recombinant protein. Following culture, the DCs were washed twice and added to 50-mL polypropylene tubes. The cationic lipid DOTAP (Boehringer Mannheim Biochemicals, Indianapolis, Ind.) was used to deliver the SP17 recombinant protein. Briefly, the recombinant protein was mixed with the lipid at room temperature for 20 min and added to DC at 37 °C in an incubator with occasional agitation for 3 hours. The cells were washed twice before being used as antigen presenting cells [26].

### Stimulation of SP17-specific CTL

Fresh PBMCs were co-cultured with antigen-pulsed DC at a ratio of 10:1 in RPMI-1640 supplemented with 10% autologous serum, IL-2 (10 IU/mL) and IL-7 (5 ng/mL) and incubated at 37 °C. IL-2 was added to the culture every 3 days thereafter. Irradiated autologous PBMC feeder cells and SP17 recombinant protein (50 mg/mL) was added to the culture every week. The cells were harvested after four rounds of stimulation and used for cytotoxicity assays [26].

### Cytotoxicity assay

A standard 4-hour LDH release cytotoxicity assay (LDH Release Assay Kit, Promega, USA) was performed to evaluate the cytotoxic activity of the SP17-stimulated T cells. Cytotoxicity against autologous breast cancer primary cells was determined at various effector:target cell ratios. For the measurement of CTL-mediated lysis of autologous lymphoblastoid cells (LCL) pulsed with SP17 or HPV16-E6 antigen, cytotoxicity assay was performed with 20:1 effector:target ratio. LCL were generated from autologous PBMCs as described previously [26]. To determine HLA restricted response, a cytotoxicity assay was performed with or without 25 μg/mL HLA-I or HLA-II (W6/32 or L243 monoclonal antibody, respectively, BioLegend 11080 Roselle Street, San Diego, CA) blocking antibodies (effector:target ratio 20:1). To block the perforin-mediated cell lysis, 5 μg/mL concanamycin A (CMA, Sigma-Aldrich, USA) was added to the co-cultures [27].

## Abbreviations

HNSCC: Head and Neck Squamous Cell Carcinoma
SP17: Sperm Protein 17
Ab: antibody
DC: Dendritic Cell
CTL: Cytotoxic T Lymphocyte
DOTAP: 1,2-di-(9Z-octadecenoyl)-3-trimethylammonium-propane
GM-CSF: Granulocytes Macrophages Colony Stimulating Factor
IL-4: Interleukin 4
PBMC: Peripheral Blood Mononuclear Cell
